# Colonic Injuries Induced by Inhalational Exposure to Particulate‐Matter Air Pollution

**DOI:** 10.1002/advs.201900180

**Published:** 2019-04-09

**Authors:** Xiaobo Li, Jian Cui, Hongbao Yang, Hao Sun, Runze Lu, Na Gao, Qingtao Meng, Shenshen Wu, Jiong Wu, Michael Aschner, Rui Chen

**Affiliations:** ^1^ Key Laboratory of Environmental Medicine Engineering Ministry of Education School of Public Health Southeast University Nanjing 210009 China; ^2^ Center for New Drug Safety Evaluation and Research China Pharmaceutical University Nanjing 211198 China; ^3^ Jiangsu Key Laboratory for Bioresources of Saline Soils Jiangsu Synthetic Innovation Center for Coastal Bioagriculture Yancheng Teachers University Yancheng 224002 China; ^4^ Department of Molecular Pharmacology Albert Einstein College of Medicine Forchheimer 209, 1300 Morris Park Avenue Bronx NY 10461 USA; ^5^ Institute for Chemical Carcinogenesis Guangzhou Medical University Guangzhou 511436 China

**Keywords:** colon, curcumin, inflammation, particulate matter

## Abstract

Particulate matter (PM) exposure has been associated with intestinal disorders. Therefore, there is an urgent need to understand the precise molecular mechanism involved and explore potential prevention strategies. In this study, inhaled PM is shown to activate inflammatory pathways in murine colon. In a panel study, it is found that ambient PM levels are significantly associated with elevated number of fecal white blood cells in healthy subjects. Acting as a promoter, PM exposure accelerates chemical carcinogenesis‐induced colonic tumor formation in a murine model. Mechanistically, RNA‐seq assays suggest activation of phosphoinositide 3‐kinase (PI3K)/AKT cascades in chronically PM‐exposed human colon mucosal epithelial cells. Ablation of up‐stream driver fibroblast growth factor receptor 4 (FGFR4) effectively inhibits inflammation and neoplasia in PM‐exposed murine colons. Notably, dietary curcumin supplement is shown to protect against PM‐induced colonic injuries in mice. Collectively, these findings identify that PM exposure accelerates colonic tumorigenesis in a PI3K/AKT‐dependent manner and suggests potential nutrient supplement for prevention.

## 1 Introduction

Epidemiologic studies have shown an association between particulate matter (PM) and an increased number of adverse intestinal outcomes. The bulk of PM enters the body majorly through inhalation, and most particles are taken up by alveolar macrophages, and ultimately excreted in the feces,^1^ suggesting potential contact of the intestinal tract with PM. Urban PM exposure resulted in increased intestinal permeability and gastrointestinal epithelial cell death in mice.^2^ It has been observed in mice that ingestion of PM‐contaminated food modified gut microbial form and function, resulting in an inflammatory response.^3^ These findings establish that the GI tract might be one of the major targets of PM exposure.

Inflammation is associated with PM‐induced intestinal disorders. Epidemiologic studies support the association between PM exposure and increased risk of appendicitis and hospitalization in patients with inflammatory bowel disease (IBD).^4^, ^5^ Ananthakrishnan et al. reviewed the environmental triggers of IBD, suggesting that PM or its components may alter the mucosal defense of the host and trigger immune responses.^6^ In addition, activation of key inflammatory pathways in multiple organs, such as NF‐κB, JNK, MAPK,^7^ Nrf2^8^ and STAT3,^9^ have been associated with PM exposure.

Inflammation in response to environmental stress is important for wound healing, while can also promote tumorigenesis of colorectal cancer (CRC). For instance, chronic mucosal inflammation and tissue damage predispose patients to the development of CRC.^10^ The four‐stage colitis‐driven tumor progression model, based on the intraperitoneal administration of mutagenic agent azoxymethane (AOM) and three cycles of inflammatory agent dextran sodium sulfate (DSS) administration, is a typical rodent CRC model designed to resemble chronic inflammation‐associated tumor progression.^11^ Since the link between PM and intestinal inflammation, and the link between chronic intestinal inflammation and CRC have been clearly demonstrated, we hypothesized that chronic PM exposure‐induced intestinal inflammation plays a key role in the increased incidence of CRC.

In this study, we evaluated pathological alterations in murine colons following chronic PM exposure, as well as potential rescue by curcumin. Subsequently, modulations in RNA expression profiles in chronic PM‐exposed human colon mucosal epithelial cells were investigated with the RNA‐seq assay. Our findings revealed that the potential molecular pathway/s involved in the adverse colonic outcomes in response to chronic PM exposure.

## 2 Results

### 2.1 Ambient PM Were Associated with Increased Number of Fecal WBC

**Figure 1:**
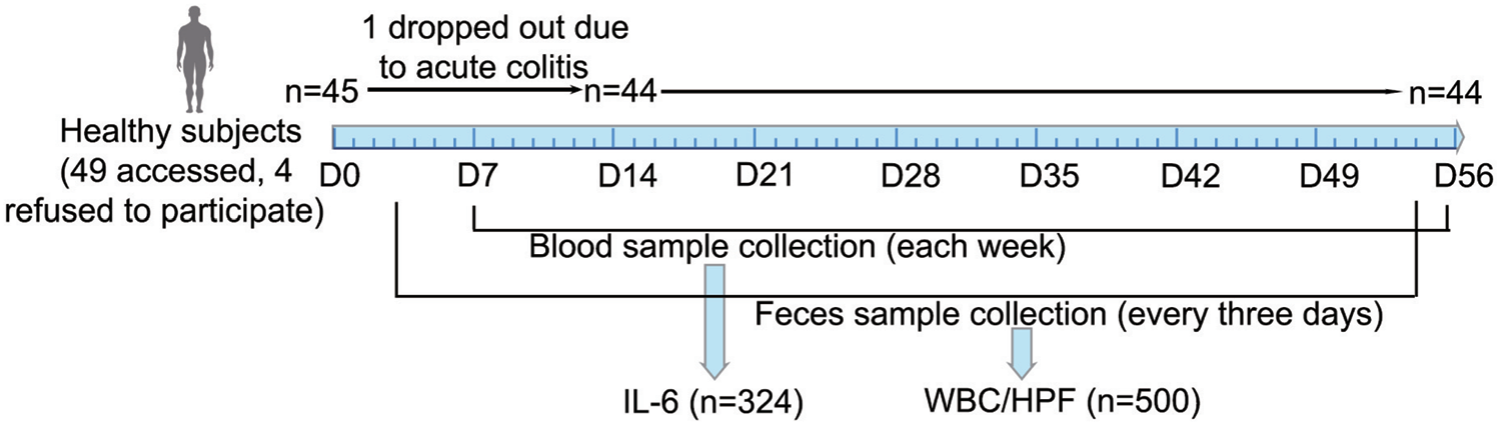
Scheme for the panel study to assess the association between air pollutant exposure and inflammatory response.

A panel study was conducted to explore the association between air pollutants exposure and inflammatory response. A total of 49 participants (26 males and 23 females) were recruited, and 44 (24 males and 20 females) completed the study (**Figure** **1**). The demographic characteristics of the participants including age, body mass index (BMI), plasma IL‐6 levels, and the number of white blood cells (WBC)/high power field (HPF) in feces are summarized in Table S1 (Supporting Information). Table S2 (Supporting Information) summarizes the statistics on six major air pollutants throughout the follow‐up period. The average concentrations of PM_2.5_ and PM_10_ during the panel study period were 84.1 and 126.7 µg m^−3^, respectively, which greatly exceeded the World Health Organization (WHO) standards (10 and 20 µg m^−3^, respectively) or China Class II standard (35 and 70 µg m^−3^, respectively). Simple correlation analyses revealed the PM_2.5_ levels were correlated with the levels of PM_10_, SO_2_, and CO (*P* < 0.0001), but not O_3_ (Table S3, Supporting Information). The levels of PM_10_ were positively correlated with the levels of NO_2_, SO_2_, and CO (*P* < 0.0001) (Table S3, Supporting Information).

Associations of plasma IL‐6 levels or numbers of WBC/HPF in feces with concentrations of air pollutants were analyzed. Results of the single‐pollutant model revealed that the levels of PM_2.5_, PM_10_, SO_2,_ and CO were positively associated with elevated numbers of WBC/HPF in feces. Levels of O_3_ were negatively associated with elevated numbers of WBC/HPF in feces (Table S4, Supporting Information). No association between concentrations of air pollutants and plasma IL‐6 levels was identified (data not shown). The levels of PM_2.5_ and PM_10_ were consistently associated with increased numbers of WBC/HPF in feces with the moving average concentrations from lag 0–4 to lag 0–8 d. The highest estimated effects of both PM_2.5_ and PM_10_ exposure on WBC/HPF in feces were noted following a 7 d exposure in which an IQR increase of PM_2.5_ (83 µg m^−3^) or PM_10_ (97 µg m^−3^) was associated with increases of 1.17 (95% CI: 0.23 to 2.81) or 1.48 (95% CI: 0.33 to 3.62) WBC/HPF in feces (Table S4, Supporting Information).

In the subsequent sensitivity analysis, after adjusting for SO_2_, NO_2_, CO, and O_3_, the estimated effects of lag 0–7 d moving average concentration of PM_2.5_ were robustly associated with elevated WBC/HPF in feces (**Table** **1**). Analogous estimated effects of PM_10_ have been identified in the sensitivity analysis, and after controlling for NO_2_, there was no significant association between PM_10_ concentration and the number of WBC/HPF (Table 1). Taken together, the results noted above emphasize the effects of consecutive PM exposure on elevated number of WBC/HPF in feces.

**Table 1:** Cumulative increases in WBC/HPF measures associated with an IQR increase in PM concentration over lags of 0–7 d

PM_2.5_ a)			PM_10_ a)		
Pollutant	WBC/HPF(95% CI)	*P* value	Pollutant	WBC/HPF(95% CI)	*P*‐value
	1.12 (0.23 to 2.81)	**0.0077**		1.48 (0.33 to 3.62)	**0.0046**
+SO_2_	0.39 (0.05 to 0.83)	**0.0212**	+SO_2_	0.34 (0.02 to 0.77)	**0.0369**
+NO_2_	0.09 (0.03 to 0.15)	**0.0022**	+NO_2_	0.01 (−0.28 to 0.41)	0.9696
+CO	0.01 (0.00 to 0.02)	**0.0121**	+CO	0.01 (0.00 to 0.01)	**0.0235**
+O_3_	0.82 (0.26 to 1.62)	**0.0220**	+O_3_	0.39 (0.10 to 0.77)	**0.0067**

^a)^ after controlling for gaseous pollutants when using two‐pollutant models.

### 2.2 Chronic PM Exposure Induced Inflammation in Murine Colons

**Figure 2:**
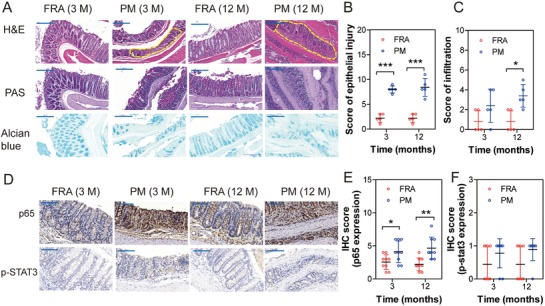
Chronic PM exposure induces chronic inflammation in murine colon. A) Representative images of colonic sections stained by H&E, PAS, and Alcian blue. Epithelial injury and inflammatory infiltration were highlighted with yellow broken line border. B) Score of epithelial injury increased in PM‐exposed colonic tissues compared to the filtered room air (FRA) group (*n* = 6, two‐way ANOVA). C) Score of inflammatory infiltration increased in PM‐exposed colonic tissues compared to the filtered room air (FRA) group (*n* = 6, two‐way ANOVA). D) Representative images of p65 and p‐Stat3 expression in colonic tissues by IHC. E) IHC score of p65 expression was calculated by two‐way ANOVA, (*n* = 9 (three nonoverlapping HPFs per mouse)). F) IHC score of p‐STAT3 expression was calculated by two‐way ANOVA, (*n* = 9 (three nonoverlapping HPFs per mouse)). M: month. *P < 0.05, ^**^ *P* < 0.01, ^***^ *P* < 0.001.

Given the newly discovered role of PM exposure in regulating intestinal disorders, mice were continuously exposed to PM for 12 months, and pathologic alterations in colonic tissues were examined at the end of each month. PM exposure exerted time‐dependent effects on the murine colon. Sporadic epithelial lesions could be observed in murine colons following 1 month of PM exposure, and more than half of the murine colons (4 of 6) showed discrete epithelial lesions and sporadic inflammatory infiltration following 3 months of PM exposure, but not in the control group (**Figure** **2**A). At the end of 12 months, epithelial lesions and the confluence of inflammatory cells were observed in all of the PM‐exposed murine colons, but only one of six in the control group. Alcian blue and PAS staining demonstrated the depletion of acidic mucins (sulfated and carboxylated) and neutral mucins in glands (Figure 2A). The epithelial injury score and inflammatory cell infiltration score were evaluated and are shown in Figure 2B,C, suggesting a significant increase in colonic injuries following PM exposure.

Compared with FRA group, levels of proinflammatory cytokines IL‐1β, but not IL‐6 and TNF‐α in serum were significantly increased following 2 or 10 months of PM exposure (Figure S1A–C, Supporting Information). Analysis of transcripts revealed local inflammation responses in the colons, showing significant upregulation of key molecules in inflammatory pathways (Stat3 and p65) in both 3‐ and 12‐month‐PM‐exposed murine colons (Figure S1D,E, Supporting Information) compared with the filter room air (FRA) exposed group. However, IHC analysis only suggested increased protein expression levels of p65 (Figure 2D–F).

### 2.3 PM Exposure Accelerated Tumorigenesis of CRC in Mice

**Figure 3:**
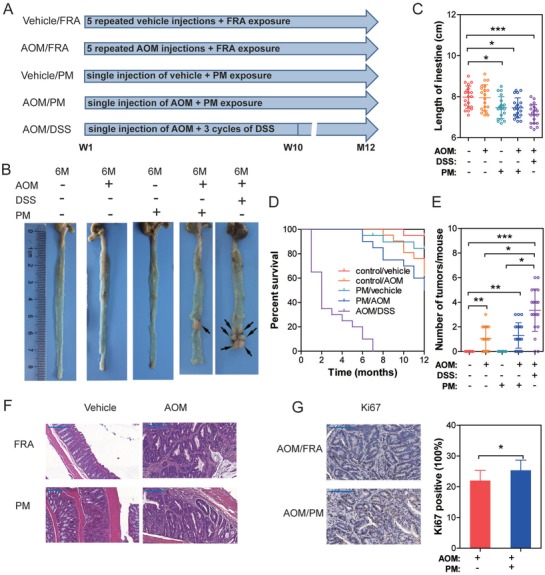
PM exposure accelerates tumorigenesis in AOM‐induced murine CRC model. A) Scheme for the experimental course of colonic tumor progression. B) Representative segments of colon from mice following FRA or PM exposure. Macroscopic tumors are shown by arrows. C) Length of murine colonic tissues (*n* = 20, one‐way ANOVA followed by Tukey's test,). D) KM plot shows the overall survival of mice with varied exposure (*n* = 20, Log‐rank test, *P* < 0.001). E) Number of macroscopic tumors per mouse (*n* = 20, Kruskal‐Wallis test followed by Dunn's test). F) H&E staining of representative colonic and tumor tissues from mice. G) Frequency of Ki67^+^ cells in tumors. (*n* = 9, three nonoverlapping HPFs per mouse, unpaired *t* test). * *P* < 0.05, ** *P* < 0.01, *** *P* < 0.001.

A model of chronic colitis‐driven tumor progression was examined, in which intraperitoneal injection of AOM is followed by 12 months of continuous PM exposure to mimic DSS‐induced colitis (**Figure** **3**A). The four‐stage colitis associated colonic cancer model (AOM‐DSS model) was used as positive control.

Corroborating previous studies, AOM/DSS‐induced colonic tumor formation was observed 3 months after AOM injection.^11^ With respect to the other four groups, no tumorigenesis was observed at the same time‐point (data not shown). Repeated intraperitoneal injections of AOM induced CRC were observed 8 months after the first AOM administration (AOM/filtered room air (FRA) group).^11^ Tumor development was accelerated in the AOM/PM treated group compared to the vehicle/FRA, AOM/FRA, and vehicle/PM groups, where the earliest tumor formation was seen following 6 month AOM/PM administration (Figure 3B).

Compared with the vehicle/FRA group, the length of vehicle/PM, AOM/PM, and AOM/DSS treated colorectal tissues were significantly reduced (Figure 3C). The overall survival was decreased and the number of tumors was increased in AOM/PM‐treated mice compared to the vehicle/PM and vehicle/FRA groups (Figure 3D,E). Histopathological sequelae of tumors was analogous in the AOM and AOM/PM‐treated mice (Figure 3F). However, an increased number of Ki67+ cells was observed in AOM/PM‐induced cancer tissues, corroborated by elevated counts of cell proliferation (Figure 3G). Accordingly, PM exposure led to accelerated and increased tumorigenesis in a chemical carcinogen model of AOM‐induced CRC.

### 2.4 Chronic PM Exposure Promoted the Neoplastic Capacity of NCM460 Cells

**Figure 4:**
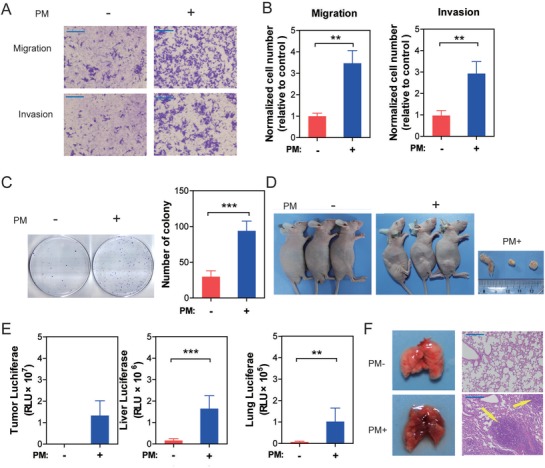
Chronic exposure to PM‐induced carcinogenesis in NCM460 cells in vitro. A) Transwell assays were used to evaluate the migration and invasion capacities of NCM460 cells. B) The migration and invasion capacities of NCM460 cells were significantly increased following PM exposure (*n* = 3, unpaired *t* test). C) The number of NCM460 cell colonies was significantly increased following PM exposure (*n* = 6, unpaired *t* test). D) PM exposure‐induced tumorigenesis of NCM460 cells in vivo. E) The metastatic potential to liver and lung for flank tumors in mice was significantly increased (*n* = 6, unpaired *t* test). F) Tumors were observed in lung tissues of mice through tail vein injection of PM‐exposed NCM460 cells (indicated by arrows). ^**^ *P* < 0.01, ^***^ *P* < 0.001.

To determine the effects of chronic PM administration on normal human colon mucosal epithelial cells (NCM460), NCM460 were maintained in control or 100 µg mL^−1^ PM for 48–72 h per passage and this process was continued for 30 passages. Then, the migration and invasion capacities of NCM460 cells were evaluated. Relative to the control, migration through transwells and invasion through Matrigel of NCM460 cells were significantly enhanced by chronic PM exposure (**Figure** **4**A,B). A foci formation assay also suggested a significant increase in colony formation in PM‐exposed cells compared to the control (Figure 4C). In vivo tumorigenesis of NCM460 cells was next examined by subcutaneous injection in nude mice. No macroscopic tumor formation was evident in control cell‐injected nude mice, but subcutaneous tumor formation was inherent to the PM‐exposed cells injected nude mice (Figure 4D). The metastatic potential for flank tumors in vivo was evaluated with luciferase biochemical assays. Significantly enhanced luciferase activities were noted in murine livers and lungs upon PM exposure compared to the control (Figure 4E), suggesting increased liver and lung metastatic burdens. PM‐exposed NCM460 cells were injected through the tail vein to observe the neoplasia in murine lungs. As shown in Figure 4F, massive macroscopic tumors were seen in murine lungs, confirmed by H&E staining (shown by white arrow). These data indicate that chronic PM exposure was involved in the neoplastic and metastatic capacity of NCM460 cells.

### 2.5 PI3K/AKT Pathway in Involved in PM‐Promoted Colonic Neoplasia

**Figure 5:**
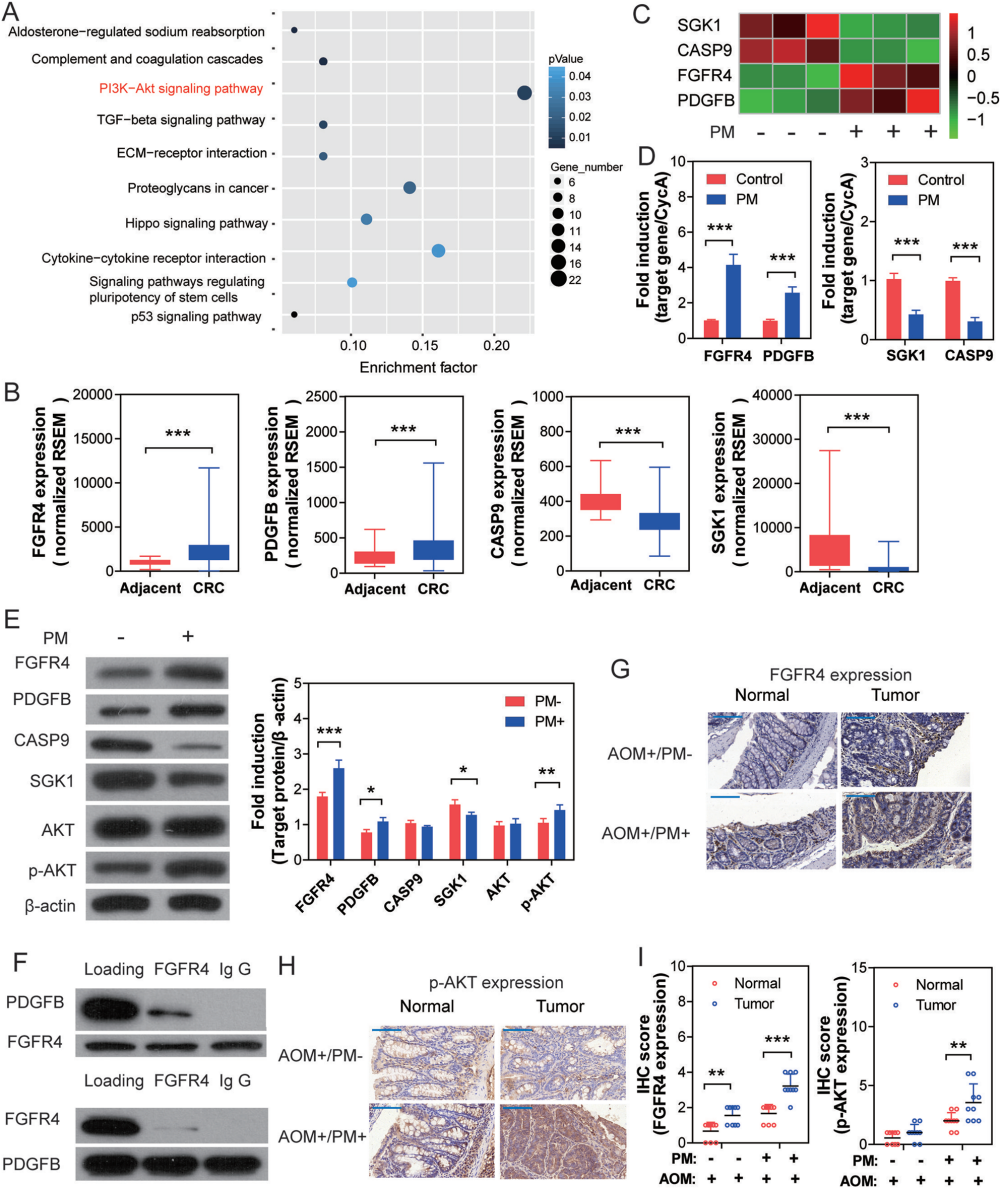
PI3K/AKT pathway plays a key role in PM exposure‐involved colonic tumorigenesis. A) KEGG enrichment of differentiated expressed genes in chronic PM‐exposed NCM460 cells. B) Aberrant expression of PI3K/AKT pathway genes in CRC. Data were retrieved from the TCGA database (data were shown as Whiskers (min to max), *n* = 41 for adjacent, *n* = 286 for CRC, Mann‐Whitney test). C) Heat map of significantly modulated genes involved in the PI3K/AKT pathway. D) Gene expression levels in NCM460 cells were validated by qRT‐PCR (*n* = 6, unpaired *t* test). E) Protein expression levels in NCM460 cells were analyzed by Western blot. (*n* = 3, unpaired *t* test) F) PPI assay suggests interaction between PDGFB and FGFR4; subsequent CO‐IP assays confirmed that FGFR4 plays a predominant role in this interaction. G) anti‐FGFR4 staining of representative tumor and paired adjacent normal tissue from murine colons. H) anti‐p‐AKT staining of representative tumor and paired adjacent normal tissue from murine colons. I) The *P*‐value of protein expression levels were calculated by two‐way ANOVA. (*n* = 9 (three nonoverlapping HPFs per mouse)). *P < 0.05, **P < 0.01, ***P < 0.001.

To further explore the differentiated gene expression profiles induced by chronic PM exposure, total RNA was extracted from control and PM‐exposed NCM460 cells for RNA‐seq analysis. The heatmap showed that a total of 496 mRNAs were differentially expressed between the two groups, including 237 upregulated and 259 downregulated genes. The cutoff was set as a fold change >1.5 and *P* < 0.05. Subsequently, these differentially expressed genes (DEGs) were subjected to KEGG pathway enrichment analysis. The PI3K/AKT pathway hit the highest DEG numbers (22 DEGs) (Table S5, Supporting Information) and the *P*‐value of pathway enrichment was 0.011 (**Figure** **5**A). Further, data retrieved from the TCGA database suggest significantly higher expression levels of fibroblast growth factor receptor 4 (FGFR4) and platelet‐derived growth factor subunit B (PDGFB), as well as lower levels of caspase 9 (CASP9) and serum‐ and glucocorticoid‐regulated kinase (SGK1) in CRC than adjacent normal tissues (Figure 5B), of which the adjusted trends were consistent with RNA‐seq profile (Figure 5C). The expression levels of these four genes were further validated by qRT‐PCR in control or PM‐exposed NCM460 cells, and the results were consistent with the RNA‐seq profile (Figure 5D).

Protein expression levels of PI3K/AKT pathway‐regulated genes were further validated by western blot analysis. As shown in Figure 5E, expression levels of FGFR4, PDGFB, and p‐AKT were significantly enhanced, while SGK1 were significantly decreased in PM‐exposed NCM460 cells compared to control, suggesting activation of PI3K/AKT signaling cascades. Protein–protein interaction (PPI) analysis was performed with the on‐line tool DAVID Bioinformatics Resources 6.8, suggesting potential physical interaction between FGFR4 and PDGFB. Therefore, the CO‐IP assay was used to confirm the interaction between FGFR4 and PDGFB, and suggested that the former one plays a predominant role in this interaction (Figure 5F). As shown in the in vivo study, enhanced expression of FGFR4 and p‐AKT were seen in AOM/PM‐induced CRC tissues compared to the adjacent normal tissue (Figure 5G–I). Together, the results suggest that the PI3K/AKT pathway plays a key role in PM exposure‐involved CRC tumorigenesis.

### 2.6 Conditional Fgfr4‐Deficient Mice Resisted PM Exposure‐Induced Colonic Inflammation

Since enhanced FGFR4 expression has been identified in PM‐exposed murine colonic, next, we generated conditional intestinal Fgfr4^−/−^ mice by crossing Fgfr4^fl/fl^ mice and mice expressing intestinal specific Cre‐recombinase under the control of the villin promoter. Initially, the pathological alterations were assessed in Fgfr4^−/−^ mice following 12‐month PM exposure. Compared with WT, there was no obvious epithelial injury, mucin depletion, and inflammatory cell infiltration observed in murine colons (Figure S2A–C, Supporting Information). FGFR4 and p65 expression levels were significantly increased in PM‐treated murine colons in WT mice, compared to KO. Furthermore, FGFR4 expression was absent in Fgfr4^−/−^ mice, and p65 expression levels were analogous in FRA or PM‐treated murine colons (Figure S2D–F, Supporting Information). Collectively, these results suggest that ablation of FGFR4 can inhibit PM‐induced inflammation in murine colon.

### 2.7 Fgfr4‐Deficient Mice Are Protected Against Experimental CRC Induced by AOM/PM Exposure

**Figure 6:**
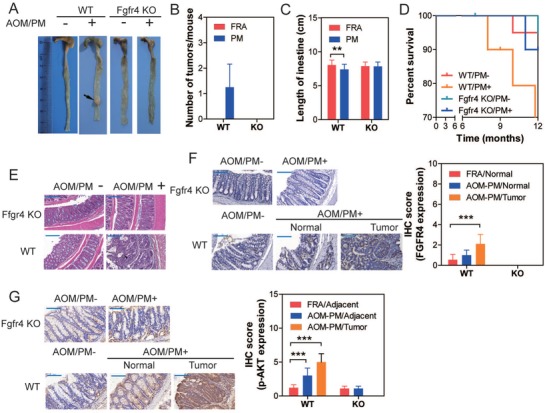
Fgfr4^−/−^ mice are resistant to PM‐induced colonic carcinogenesis. A) Representative segments of colon from mice following FRA or PM exposure. Macroscopic tumors are indicated by arrows. B) Number of macroscopic tumors per mouse (*n* = 20). C) Length of colonic tissues (*n* = 20, two‐way ANOVA). D) KM plot demonstrates the overall survival of mice with different treatments (*n* = 20, Log‐rank test, *P* < 0.05). E) H&E staining of representative colon or tumoral tissues from mice. F) anti‐FGFR4 staining of representative colon and tumoral tissues from mice. The *P* values of IHC score were calculated by two‐way ANOVA. (*n* = 9 (three nonoverlapping HPFs per mouse)). G) anti‐p‐AKT staining of representative colon and tumoral tissues from mice. The *P* values of IHC score were calculated by two‐way ANOVA (*n* = 9 (3 nonoverlapping HPFs per mouse)). ^**^ *P* < 0.01, ^***^ *P* < 0.001.

FGFR4 is highly expressed in CRC tissues and has been suggested to play a key role in the development of various cancers.^12^, ^13^ Therefore, the role of FGFR4 in the development of AOM/PM‐induced CRC was evaluated. Following analogous AOM/PM administration protocol (see above), the tumor number was significantly lower in the CRC tissues in Fgfr4^−/−^ compared to WT mice (**Figure** **6**A,B). Furthermore, the length of colorectal tissues and overall survival were significantly rescued in Fgfr4^−/−^ compared to WT mice (Figure 6C,D). There was no tumor formation observed in colonic tissues of AOM/PM treated Fgfr4^−/−^ mice by histological analysis (Figure 6E). IHC staining demonstrated depletion of FGFR4 expression in colonic tissues of Fgfr4^−/−^ mice and significantly increased FGFR4 expression in tumor tissue of AOM/PM‐treated WT mice, compared with normal colon of FRA treated WT mice. (Figure 6F). Furthermore, p‐AKT expression levels in colons of AOM/PM‐administrated Fgfr4^−/−^ mice were analogous to those of AOM/FRA‐treated KO mice (Figure 6G).

Corroborating the in vivo study, stable FGFR4 KO NCM460 cells were set up through lentivirus infection, WT and FGFR4 KO NCM460 cells were both exposed to PM for 30 passages. The capacities for migration and invasion, as well as the subcutaneous and pulmonary tumor formation were significantly inhibited in FGFR4 KO cells compared to WT NCM460 cells (Figure S3, Supporting Information). Collectively, these results suggest that FGFR4 is an upstream driver in triggering the PM‐induced neoplasia, both in vivo and in vitro.

### 2.8 Curcumin Ameliorates Chronic PM Exposure‐Induced Inflammation in Murine Colons

Lewis and Abreu reviewed several diet supplements that protect against intestinal inflammation or increase the efficacy of inflammatory bowel disease therapy, such as omega‐3 long‐chain fatty acids, vitamin D, curcumin, and soluble fiber.^14^ However, the protective effects of these diet supplements against PM‐induced colonic disorders remain unknown. Therefore, it was necessary to evaluate their effects on long‐term PM‐treated NCM460 cells. We found that only curcumin (20 × 10^−6^ м) administration completely rescued FGFR4, PDGFB, CASP9, and SGK1 gene expressions in PM‐treated NCM460 cells to levels indistinguishable to those in control levels (Figure S4A, Supporting Information). DHA treatment partially rescued FGFR4 expression levels in NCM460 cells to control (Figure S4B, Supporting Information), while 1α25 (OH_2_) D_3_ did not modulate gene expression levels (Figure S4C, Supporting Information).

**Figure 7:**
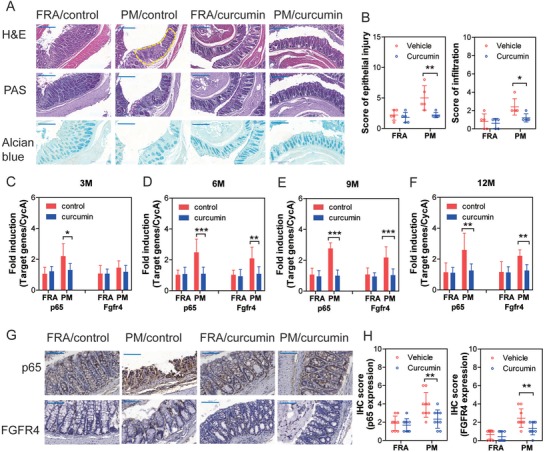
Curcumin protects against chronic PM exposure‐induced colonic inflammation in mice. A) Representative images of colonic sections stained by H&E, PAS, and Alcian blue following 12‐months PM exposure. The epithelial injury and inflammatory infiltration area in colonic epithelia were highlighted with yellow broken line border. B) Score of epithelial injury and inflammatory infiltration increased in PM‐exposed colon compared to the filtered room air (FRA) group (*n* = 5, two‐way ANOVA). C) mRNA expression levels of p65 and Fgfr4 in murine colonic tissues following 3‐month PM exposure (*n* = 6, two‐way ANOVA). D) mRNA expression levels of p65 and Fgfr4 in murine colons following 6‐month PM exposure (*n* = 6, two‐way ANOVA). E) mRNA expression levels of p65 and Fgfr4 in murine colons following 9‐month PM exposure (*n* = 6, two‐way ANOVA). F) mRNA expression levels of p65 and Fgfr4 in murine colons following 12‐month PM exposure (*n* = 6, two‐way ANOVA). G) Representative images of anti‐p65 and anti‐FGFR4 staining of murine colons following 12 months PM exposure. H) The IHC score of p65 and FGFR4 (two‐way ANOVA, *n* = 9 (three nonoverlapping HPFs per mouse)) *P < 0.05, **P < 0.01, ***P < 0.001.

Treatment with curcumin improved the histopathologic sequelae of murine PM exposure‐induced colonic inflammation (**Figure** **7**A,B). The anti‐inflammatory effects of curcumin are attributable in part to inhibition of NF‐κB.^15^ Consistent with these findings, p65 and Fgfr4 mRNA were attenuated in colonic mucosa in the PM/curcumin‐treated group (Figure 7C–F). Protein expression levels of p65 and FGFR4 in colonic epithelia were also suppressed in the PM/curcumin‐treated group (Figure 7G,H), compared with vehicle/PM‐treated group. Our findings establish that oral supplementation with curcumin ameliorated PM‐induced colonic inflammation. Accordingly, curcumin might serve as a potential candidate for prevention and treatment of PM‐induced colonic disorders.

## 3 Discussion

In this study, we showed that PM exposure accelerated AOM‐induced experimental colonic tumorigenesis in mice. Mechanistically, FGFR4‐triggered activation of the PI3K/AKT pathway played a key role in the PM‐accelerated progression of colonrectal tumor formation.

Recent publications have established an association between PM exposure and intestinal disorders, yet the underlying mechanisms predominantly refer to alterations in the intestinal microenvironment, including increased inflammation, oxidative stress, and disturbance of the intestinal flora or metabolism.^16^, ^17^ In addition, PM has been reported as one of the important environmental triggers in IBD.^6^ Corroborating the results in the present study, PM exposure had been previously shown to generate oxidative stress‐dependent NF‐κB activation, in turn, disrupting tight junctions and ultimately increasing intestinal permeability in PM‐exposed mice.^2^ Furthermore, proinflammatory cytokines CXCL1, IL‐1β, and IL‐10 were significantly increased in the intestines of mice exposed to PM.^18^ In another study, lipid metabolism was disrupted by exposure to ambient ultrafine PM for 10 weeks.^19^ Results of our panel study also confirmed local intestinal inflammatory response upon PM exposure. Inflammation exerts protective properties for wound healing; however, it also promotes tumorigenesis if uncontrolled during the recovery phase.^20^ Till now, whether the PM‐induced intestinal inflammation playing a protective or deleterious role remains unclear.

Our results demonstrated signs of colonic inflammation and activation of inflammatory pathways, but no spontaneous tumorigenesis in murine colonic tissues in response to chronic PM exposure. In addition, numerous studies have demonstrated that inflammation is one of the major drivers of tumorigenesis in the colitis‐associated CRC model.^10^, ^20^ Thus, we hypothesized that PM exposure may play a role in the development of CRC by accelerating the tumorigenesis induced by mutagenic determinants, but PM may not be a direct mutagenic agent itself. Administration of the mutagenic agent AOM resulted in the development of spontaneous tumors within 30 weeks, while combined administration of AOM with the inflammatory agent DSS caused rapid growth of colon tumors within 10 weeks.^11^ Our results demonstrated that PM‐induced intestinal inflammation accelerated AOM‐induced murine CRC, strongly supporting our hypothesis.

RNA‐seq and bioinformatics analysis suggested that PM exposure‐induced activation of PI3K/AKT is mechanistically related to the acceleration of CRC formation. The dysregulation of the PI3K/AKT pathway, with copy number alteration and increased expression, is commonly observed in a number of cancers.^21^, ^22^ In addition, ambient PM exposure has been associated with the overexpression of p‐AKT in adipose tissue,^23^ testes,^24^ or lung cells.^25^The pathway can be activated upstream by a number of receptor protein tyrosine kinases and transduce downstream cell survival signals, ultimately resulting in increased cell proliferation and loss of apoptosis.^26^ In this study, it was found that FGFR4 could act upstream to activate the PI3K/AKT pathway, suppressing the expression of SGK1 and the apoptosis‐related cysteine peptidase CASP9. Consistent with these results, overexpression of FGFR4, the specific receptor for fibroblast growth factor (FGF)‐1 and FGF‐3, was associated with epithelial‐to‐mesenchymal transition, tumor progression, and drug resistance of CRC.^27^, ^28^ The results from this study demonstrated that ablation of FGFR4 effectively blocked AOM/PM‐induced experimental CRC in mice, suggesting that the PI3K/AKT cascade plays an important role in PM‐involved colonic tumorigenesis.

The turmeric‐derived phytochemical, curcumin, is well known to be beneficial in IBD via inhibition of NF‐κB, p38 mitogen‐activated protein kinase, PI3K/AKT or inflammatory cytokine expression in mice and humans.^29^, ^30^, ^31^ In line with these studies, oral administration of curcumin protected against PM exposure‐induced inflammation in murine colons affording potential rescue from PM‐induced colonic disorders.

## 4 Conclusion

Taken together, our novel results establish that the PI3K/Akt cascades mediate the colonic tissue damage and tumorigenesis of PM exposure. Furthermore, chronic PM exposure‐induced colitis accelerates AOM‐induced experimental CRC. We posit a link between PM exposures, inflammation and mutagenic agent, as well as a molecular mechanism that mediate tumor development in the colon. Upregulation of FGFR4 mediated activation of PI3K/AKT pathway is associated with PM/AOM‐induced tumorigenesis of CRC. Finally, the phytochemical, curcumin, is shown to be efficacious in protecting against colonic disorders resulting from chronic ambient PM pollution.

## 5 Experimental Section

*Experimental Design*: The goal of this study is to evaluate the role of chronic ambient PM exposure played in the elevated prevalence of adverse colonic outcomes and the potential molecular mechanism involved. It is hypothesized that chronic PM exposure triggered colonic inflammation, which played a role to accelerate mutagenic agent‐induced tumorigenesis.

*Chemicals*: Urban PM (SRM 1648a) was purchased from the National Institute of Standards and Technology (NIST), USA. The atmospheric particulate matter was collected in an urban area and their major components were investigated by a research group from NIST.^32^ DHA (D2534), vitamin D (1α25 (OH2) D3) (731 285), and curcumin (C1386) were purchased from Sigma, USA.

*Panel Study Participants and Design*: Forty‐nine healthy college students were recruited from the Xiaozhuang Campus, Nanjing Xiaozhuang University, which is in the north urban area of Nanjing, China. Criteria for exclusion included those with current antibiotics administration, and a history of tobacco smoking, regular alcohol drinking, diabetes, obesity (BMI over 30), hypertension (systolic blood pressure over 120 mm of mercury (mm Hg) and/or diastolic blood pressure over 80 mm Hg), clinically diagnosed chronic cardiovascular diseases, chronic kidney diseases, chronic gastrointestinal diseases, and respiratory diseases. This study was approved by the Institutional Review Board at the Zhongda Hospital, Southeast University. Written informed consent was obtained from all participants at enrollment.

The design consisted of a panel study with repeated measurements of ambient air pollutants, plasma IL‐6, and number of WBC/HPF in feces of enrolled individuals. For each participant, eight blood collections were scheduled on Sunday mornings from December 17, 2017 to February 4, 2018, and collected between 8:00 am to 11:00 am to control for diurnal rhythm. From December 13, 2017 to February 3, 2018, feces samples within 2 h were collected every 3 d. Data on individual demographic characteristics (gender, height, weight, and BMI) were collected at enrollment. To minimize the effects of diet on the gastrointestinal tracts, all participants were asked to refrain from overeating and barbeque during the study period. The daily activities of all participants were restrained to areas no more than 3.0 km away from the campus. A self‐administered questionnaire of daily diet, medicine administration, and information on location was completed by all participants.

*Microscopic Inspection of WBC/HPF in Feces*: Fecal samples (20–30 g) from each participant were collected every 3 d, and all feces were examined immediately under a microscope. Each specimen was separately examined by two experienced technicians, and the mean of number of WBCs were counted under 5 non‐overlapping high‐power fields.

*Ambient Air Pollutants Assessment*: The daily concentrations of six criteria pollutants, including PM_2.5_, PM_10_, nitrogen dioxide (NO_2_), ozone (O_3_), carbon monoxide (CO), and sulfur dioxide (SO_2_) were collected from the Maigaoqiao Environmental Monitoring Station of Nanjing, which is located 2.9 km away from the Xiaozhuang Campus.

*Statistical Analyses for Panel Study*: The linear mixed‐effect model was applied to evaluate the air pollutant‐plasma IL‐6 association and air pollutant‐fecal WBC association. All models were performed using SAS 9.4 software (SAS Institute, Cary, NC, USA). The significance level was set at *P* < 0.05.

In the single‐constituent model, the air pollutants were incorporated as fixed‐effect terms one at a time, and a random intercept for each subject was added to account for correlations among multiple repeated measurements collected per subject. Several covariates were included as fixed‐effect terms, such as the moving average of mean temperature and relative humidity on the present day and previous 10 d, to control for the confounding effects of weather conditions and individual characteristics including age, gender, BMI, and day of study. Study has demonstrated that associations between biological effects and cumulative exposures over multiple days are more robust than exposures over a single day.^33^ Therefore, we fitted the model using moving‐average lags from 0–1 d to a maximum lag of 0–10 d to explore the lag structures within the short‐term effects of air pollutants on plasma IL‐6 and fecal WBC number. The data of WBC numbers has been log‐transformed. For the sensitivity analysis, a “two‐pollutant model” was built to assess whether the effects of PM_2.5_ were dependent on simultaneous exposure to other criteria air pollutants.

Correlations between criteria air pollutants were analyzed by Spearman correlation. Results were expressed as estimated percent change (95% CI) in plasma IL‐6 or fecal WBC number associated with interquartile range (IQR) increases in air pollutants.

*Animals*: Male C57BL/6 mice (20–22 g) were purchased from Vital River Laboratory Animal Technology (China). Homozygous Fgfr4^fl/fl^ mice on a C57BL/6 background were generated by insertion of a loxP sequences within introns 7 and 8 of Fgfr4 gene.^34^ Mice expressing intestinal specific Cre‐recombinase under the control of the villin‐promoter (p‐Villin mouse) were purchased from Nanjing Biomedical Research Institute of Nanjing University. The conditional intestinal Fgfr4^−/−^ mice were generated by crossing Fgfr4^fl/fl^ mice and p‐Villin mice. Animals were treated humanely and all experimental protocols were approved by the Committee on Animal Use and Care of Southeast University, China. All the methods in this study were performed according to approved guidelines. Mice were bred in the specific pathogen‐free class laboratory animal housing of Southeast University. Five mice were housed in each polycarbonate cage with ad libitum access to food and water. Light cycles were set on a 12/12 h light/dark cycle, and room temperature was set at 22.5 °C.

*Animal Treatment*: Dynamic inhalation exposure chambers were outfitted with extensive air quality monitoring equipment and an aerosol generator (Beijing HuiRongHe Technology Co., Ltd, China). The concentration of suspended PM was measured gravimetrically by a real‐time dust monitor (CEL‐712 Microdust Pro, CASELLA CEL Inc., USA. Exposure was carried out in stainless steel Hinners‐type whole‐body inhalation chambers; the treatment groups received PM, and the control received high efficiency particulate air (HEPA)‐filtered room air (FRA) at the same flow rate. The mice were exposed in their respective chambers for 2 h per day (10–12 a.m.), 5 d (from Monday to Friday) per week, with a mean mass concentration of PM 0.4 mg m^−3^.

The first batch of animal experiments included two groups (with 120 C57BL/6 mice in each group): control mice exposed to FRA or mice exposed to PM. Mice were exposed to PM for 12 consecutive months. At the end of each month, blood samples of 10 mice from each group were collected from the angular vein, and then mice were sacrificed. Images of the colorectal tissues were taken, and the lengths of the colorectal tissues were recorded. Next, 4 of 10 colorectal tissues were stored in liquid nitrogen, while the other six colorectal tissues were fixed “Swiss‐roll” style in 4% paraformaldehyde (PFA) at 4 °C.

A second batch of mice were divided into five groups (with 50 C57BL/6 mice in each group): control mice treated with five repeated intraperitoneal injections of vehicle (PBS) (one injection per week) and exposed to FRA (vehicle/FRA); mice treated with five repeated intraperitoneal injections of AOM (7.5 mg kg^−1^ body weight, single injection per week) and exposed to FRA (AOM/FRA); mice treated with a single injection of vehicle (PBS) and exposed to PM (vehicle/PM); mice treated with a single injection of AOM and exposed to PM (AOM/PM); the positive control mice treated with a single injection of AOM (7.5 mg kg^−1^ body weight) and then DSS (1.5% (wt/vol) in drinking water) was administered for 5 d followed by 16 d of a water regimen for three separate cycles (AOM/DSS).^11^

A third batch of mice included four groups (with 40 mice in each group): wild type (WT) mice exposed to FRA or PM; Fgfr4^−/−^ mice exposed to FRA or PM.

A fourth batch of mice included four groups (with 50 mice in each group): wild type (WT) mice or Fgfr4^−/−^ mice treated with a single injection of vehicle (PBS) and exposed to FRA; wild type (WT) mice or Fgfr4^−/−^ mice treated with a single injection of AOM and exposed to PM.

A fifth batch of mice included four groups (with 40 C57BL/6 mice in each group): mice exposed to FRA with common or curcumin‐enriched (0.2% wt/wt) diets;^35^ mice exposed to PM with common or curcumin‐enriched (0.2% wt/wt) diets.

For the second and fourth batches of animal experiments, mice were exposed to FRA or PM for 12 consecutive months. Half of the mice from each group (20 mice) were randomly selected for observation of their overall survival for up to 12 months. Kaplan‐Meier (KM) survival plots were drawn according to the date of death of mice. For the other 30 mice from each group, the checkpoints were set from 3 to 12 months. At the end of each month, two mice were randomly selected and sacrificed for the macroscopic and histological examination of colorectal tissues. Images were taken of all colorectal tissues, and the length of the colorectum, as well as the number of tumors, were recorded. Colorectal tissues were stored in liquid nitrogen or fixed “Swiss‐roll” style in 4% PFA at 4 °C.

For the third and fifth batches of animal experiments, mice were exposed to FRA or PM for 12 consecutive months. At the end of 3, 6, 9, and 12 months, 10 mice were randomly selected and sacrificed for the macroscopic and histological examination of colorectal tissues. Colorectal tissues were stored in liquid nitrogen or fixed “Swiss‐roll” style in 4% PFA at 4 °C.

*Histopathological Analysis of Mice Lung Tissues*: PFA fixed tissues were embedded in paraffin serially sectioned (5 µm) and stained with hematoxylin and eosin (H&E). Sections were stained with Alcian blue (pH 2.5) to demonstrate acidic (sulfated and carboxylated) mucins or periodic acid Schiff (PAS) to characterize the neutral mucins, which were used to evaluate the mucodepletion in murine colon.

The epithelial injury score and inflammatory infiltration score were defined as follow: the epithelial injury score is the sum of the scores of gland mucodepletion plus scores of tissue damage, which are scored on a scale of 0 to 3 (mucodepletion: 0, none; 1, mild; 2, moderate; 3, severe; tissue damage: 0, no mucosal damage; 1, discrete epithelial lesions; 2, surface mucosal erosion or focal ulceration; 3, extensive mucosal damage and extension into deeper structures of the bowel wall).

The inflammatory infiltration score is the sum of infiltration score plus the percent area of each section. Infiltration was scored on a scale of 0 to 3 (0, no inflammatory cells; 1, infiltration around crypt bases; 2, infiltration of muscularis mucosa; 3, infiltration of submucosa), and the percent area of each section was scored on a scale of 0 to 4 (0, no involvement; 1, ≤ 25%; 2, ≤ 50%; 3, ≤ 75%; 4, ≤ 100%).

Scores were determined for the proximal, middle, and distal colon. Total scores are the sum of the scores of each segment. Histological scoring of tissues was performed by two experienced pathologists in a blinded manner.

*Immunohistochemistry Staining*: After dewaxing, immunohistochemistry staining were performed as described,^36^ and incubated overnight at 4 °C with mouse monoclonal antibodies against FGFR4 (1:50) (Abcam, USA), p‐Akt (1:100) (Cell Signaling Technology, USA), p65 (1:500) (Abcam, USA), p‐STAT3 (1:100) (Abcam, USA), Ki67 (1:100) Abcam, USA). Antibody binding to tissue sections was visualized with a biotinylated rabbit anti‐mouse IgG antibody (1:400; DAKO) and developed with diaminobenzidine (DAB) as a substrate. For the negative controls, the primary antibodies were omitted. Each section was examined under microscopy by two histologists using a semiquantitative immunoreactivity score (IRS) methods as follow: Category A documented the intensity of immunostaining as 0–3 (0, negative; 1, weak; 2, moderate; 3, strong); category B documented the percentage of immunoreactive area as 1 (0%–25%), 2 (26%–50%), 3 (51%–75%), and 4 (76%–100%). IRS resulted from the multiplication of category A and B of each high‐power field (HPF) (× 400 magnification). Each tissue condition was counted in three non‐overlapping HPFs.

*ELISA*: The levels of inflammatory cytokines in plasma were measured using a Human IL‐6 ELISA Kit (Multi Sciences, China). The levels of IL‐6, TNF‐α, and IL‐1β in murine plasma were measured by a Mouse IL‐6 ELISA kit, Mouse TNF‐alpha ELISA Kit and Mouse IL‐1β ELISA Kit (Multi Sciences, China) according to the manufacturer's instructions. Each sample was assayed in duplicate.

*qRT‐PCR Assay*: The mRNA expression levels were determined by reverse transcription of total RNA followed by qRT‐PCR analysis on a Quant Studio 6 Flex system (Applied Biosystems, Life Technologies, USA) using SYBR PCR Master Mix reagent kits (Toyobo). Primer sequences are shown in Table S6 (Supporting Information), and all experiments were performed in duplicate. The mRNA levels provided here are relative to CycA for the indicated gene.

*Expression Plasmid Construction and Stable Lentivirus Transduction*: Firefly luciferase cDNA harboring lentiviral (LTV) expression shuttle vectors was constructed and stably transduced into NCM460 cells as described.^37^

FGFR4 shRNA lentiviruses were generated by cotransfection with packaging plasmids, pSPAX2 and pMD2G. The shRNA lentivirus harbored a short‐hairpin RNA sequence to target genes. NCM460 cells were added with lentivirus (MOI = 30) and treated with Blasticidin S for 2 weeks to obtain the stable transduction into NCM460 cells.

*Chronic Treatment of NCM460 Cells with PM*: Wild type (WT) or FGFR4 KO NCM460 cells (1 × 10^6^) were seeded into 10 cm (diameter) dishes for 24 h. Cells were maintained in 0 (control) or 100 µg mL^−1^ PM for 48–72 h per passage. This process was continued for 30 passages. Total RNA and protein of control or PM treated NCM460 cells were isolated for further assays.

*Cell Migration and Cell Invasion Assays*: For cell migration assays, 1 × 10^5^ control or long‐term PM‐treated NCM460 cells were transferred into a Transwell insert, and incubated with complete medium for 24 h. For cell invasion assays, 1 × 10^5^ control or long‐term PM‐treated NCM460 cells were transferred into a Transwell insert containing wells pre‐filled with Matrigel (CytoSelect 24‐Well Cell Invasion Assay Kit; Cell Biolabs, USA) and cultured with complete medium for 48 h. Cell migration and invasion was determined from triplicates for each treatment after crystal violet staining.

*Colony Formation Analysis*: Five hundred control or PM treated NCM460 cells were seeded in 10 cm plates with six replicates and allowed to attach for 24 h. Colony number of each plate was determined as described.^37^

*In Vivo and Ex Vivo Nude Mice Flank Tumor Experiments*: Nude mice (18–20 g) were purchased from Model Animal Research Center of Nanjing University, China; maintained and used according to the guidelines of the Committee on Animal Use and Care of Southeast University. Female nude mice were injected subcutaneously on the dorsal flanks, with 5 × 10^6^ luciferase‐expressing stably transformed NCM460 cells suspended in 0.5 mL DMEM. Three weeks after injection, mice were killed under ether anesthesia. Lung, liver, and tumor tissues were removed for biochemical luciferase activity analysis. The activity of luciferase was determined by a luminometer (Sirius, Berthold Detection Systems, Germany). One piece of lung, liver or tumor was stored in PFA for histological analysis.

*DHA, Curcumin, and Vitamin D (1α25 (OH2) D3) Administration to NCM460 Cells*: Curcumin, docosahexaenoic acid (DHA), and 1α25 (OH2) D3 were prepared as stock solutions in DMSO and stored at −20 °C. For each experiment, the stock solutions were diluted with cell culture medium to obtain a final DMSO concentration of 0.5% (v/v).

The NCM460 cells (1 × 10^6^) were seeded into 10 cm (diameter) dishes for 24 h. Cells were divided into eight groups: control (PBS)/vehicle (0.5% (v/v) DMSO) treated cells; PM (100 µg mL^−1^ PM) /vehicle treated cells; control/curcumin (20 × 10^−6^ m) treated cells; PM/curcumin treated cells; control/DHA (50 × 10^−6^ m) treated cells; PM/DHA treated cells; control/vitamin D (100 × 10^−9^ m) treated cells; PM/vitamin D treated cells. All of the cells were maintained for 48–72 h per passage, and this process was continued for 30 passages. Total RNA and protein of control or long‐term PM treated NCM460 cells were isolated for further assays.

*RNA‐Seq Assays and Functional Analysis of Modulated RNA*: Total RNA from control or long‐term PM treated NCM460 cells was purified and processed using an Illumina TruSeq RNA sample prep kit (Illumina, USA). Pooled RNAseq libraries were sequenced by an Illumina HiSeq 2000 sequencer. Alignments were parsed using the Tophat program and differential expressions were determined through DESeq/DEGseq. The Benjamini‐Hochberg procedure was used to correct for hypothesis testing, with an FDR cutoff of 0.05 for collected data. KEGG enrichment over differentially expressed genes was performed using a functional annotation tool, Database for Annotation, Visualization, and Integrated Discovery (DAVID 6.7).

*Western Blot Assay*: Protein expression levels were analyzed by immunoblotting with primary antibodies FGFR4 (1:50 dilution; Abcam), PDGFB (1:1 000 dilution; Abcam), CASP9 (1:1 000 dilution; Cell Signaling Technology, USA), SGK1 (1:1 000 dilution; Cell Signaling Technology, USA), Akt (1:1 000 dilution; Cell Signaling Technology, USA), p‐Akt, (1:1 000 dilution; Cell Signaling Technology, USA) or β‐actin (1:10 000 dilution; Sigma, USA).

*Endogenous Protein Immunoprecipitation*: Immunoprecipitation of endogenous proteins was accomplished with a Universal CO‐IP kit (Active Motif, USA). NCM460 whole cell extracts were first incubated with protein A agarose beads. Cleared supernatants were incubated with FGFR4, PDGFB, or normal human IgG for 2 h before addition of protein A to the agarose beads. After binding, beads were pelleted by centrifugation and washed with the buffer. After washing, immunoprecipitated materials were eluted and immunoblotted with anti‐human FGFR4 (1:500 dilution) or PDGFB (1:1 000 dilution) primary antibodies.

*Statistical Analysis*: Data were presented as mean ± SD unless indicated otherwise. Data distributions were tested for normality, and variance equality between groups was assessed using the Levene's test. The significance was set at *P* < 0.05 for the data. The 2^‒ΔΔ^ *^Ct^* method was used to analyze the qRT‐PCR results. Statistical comparisons were performed using unpaired Student's *t* tests (two‐tailed), one‐way ANOVA or two‐way ANOVA. Statistical analysis was performed by SPSS (version 12.0).

## Conflict of Interest

The authors declare no conflict of interest.

## Supporting information

Supplementary
