# Correction to “Colonic Injuries Induced by Inhalational Exposure to Particulate‐Matter Air Pollution”

**DOI:** 10.1002/advs.202407554

**Published:** 2024-07-23

Li X, Cui J, Yang H, Sun H, Lu R, Gao N, Meng Q, Wu S, Wu J, Aschner M, Chen R*. Colonic Injuries Induced by Inhalational Exposure to Particulate‐Matter Air Pollution. *Advanced Science* **2019**, *6*, 1900180.

https://doi.org/10.1002/advs.201900180

In Figure 4A, the image of the Migration and Invasion of PM (‐) group appeared incorrectly. We thus request to correct Figure 4A as follow:

Corrected Figure 4A


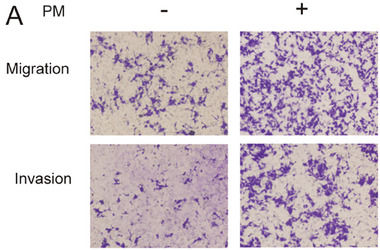


In Figure S2D, the image of p65‐WT(FRA) was used incorrectly. We request to correct Figure S2D as follow:

Corrected Figure S2D:


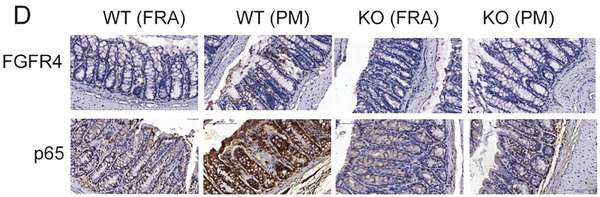


We apologize for these errors.

