# Supplementary material for: Colonic Injuries Induced by Inhalational Exposure to Particulate‐Matter Air Pollution
Source: Adv Sci (Weinh). 2019 Apr 9;6(11):1900180. doi: 10.1002/advs.201900180 (PMC6548988; doi:10.1002/advs.201900180)
Supplement: Supplementary file 1 — Supplementary [file ADVS-6-1900180-s001.pdf]

Copyright WILEY-VCH Verlag GmbH & Co. KGaA, 69469 Weinheim, Germany, 2019.

ADVANCED SCIENCE Open Access

Supporting Information

for *Adv. Sci.,* DOI: 10.1002/advs.201900180

Colonic Injuries Induced by Inhalational Exposure to Particulate-Matter Air Pollution

*Xiaobo Li, Jian Cui, Hongbao Yang, Hao Sun, Runze Lu, Na Gao, Qingtao Meng, Shenshen Wu, Jiong Wu, Michael Aschner, and Rui Chen**

Copyright WILEY-VCH Verlag GmbH & Co. KGaA, 69469 Weinheim, Germany, 2019.

Supporting Information

Inhalational Exposure of Particulate Matter Air Pollution Induced Colonic Injuries

Xiaobo Li[1], *Jian Cui*[1], *Hongbao Yang*[2], *Hao Sun*[1], *Runze Lu*[1], *Na Gao*[1], *Qingtao Meng*[1], *Shenshen Wu*[1], *Jiong Wu*[3], *Michael Aschner*[4], *Rui Chen*[1, 5]*

Figure S1 Inflammatory response induced by PM exposure. A: Plasma levels of IL-6 B: TNF-α and C: IL-1β (mean ± SE, *n*=3, two-way ANOVA followed by Bonferroni post hoc). D: mRNA expression levels of p65; E: Stat3, F: Relb and G: Nrf2 in murine colonic tissues (*n*=8, two-way ANOVA). $^{*}P$<0.05, $^{**}P$<0.01, $^{***}P$<0.001

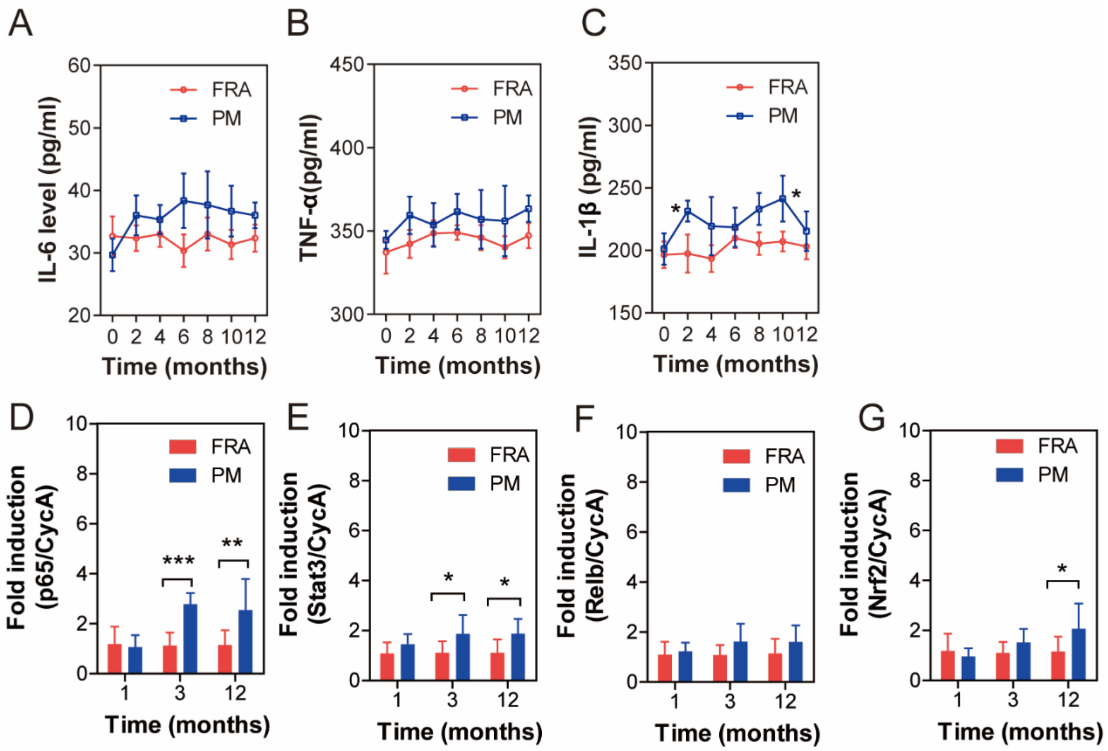

Figure S2: Fgfr4$^{-/-}$ mice are resistant to PM exposure-induced colonic inflammation.

A: Representative images of colonic sections stained by H&E, PAS and Alcian blue following 12-months of PM exposure. The epithelial injury and inflammatory infiltration area was highlighted with yellow broken line border. B: Score of epithelial injury and C: inflammatory infiltration increased in PM-exposed colonic tissues (*n*=5, two-way ANOVA). D: Representative images of anti-FGFR4 and anti-p65 staining of murine colons following 12 months of PM exposure. E: The IHC score of FGFR4 and F: p65 expressions. (*n*=9 (3 non-overlapping HPFs per mouse), two-way ANOVA) *** *P*<0.001

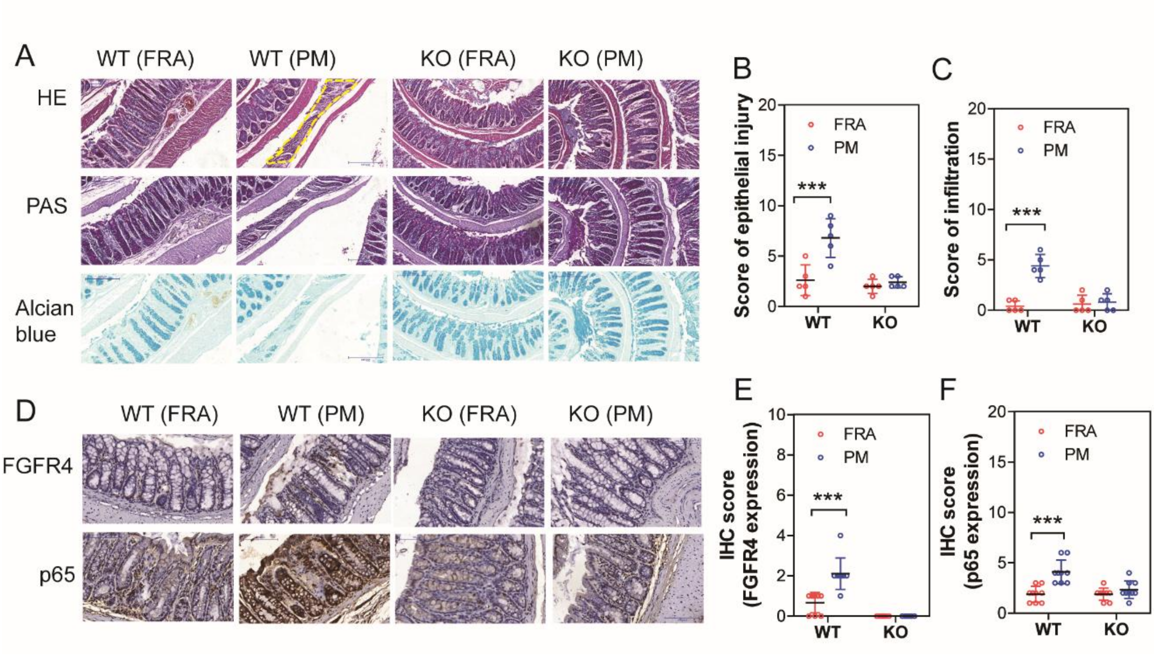

Figure S3 FGFR4 is an up-stream driver of PM exposure-induced neoplasia

A: Inhibition of FGFR4 expression significantly decreased number of colonies of NCM460 cells (*n*=6, unpaired *t* test). B: Representative images of Transwell assays. C: The migration and invasion capacities of NCM460 cells were significantly decreased following inhibition of FGFR4 expression levels (*n*=3, Wilcoxon rank sum test). D: Suppression of FGFR4 decreased the volume of subcutaneous tumors. E: The metastatic potential to liver and lung for flank tumors in mice were significantly decreased (*n*=6, unpaired *t* test). F: Representative images of H&E staining on lung tissue sections from mice injected with NCM460 cells (infected with or without FGFR4 siRNA) through tail vein. * *P*<0.05, ** *P*<0.01, *** *P*<0.001

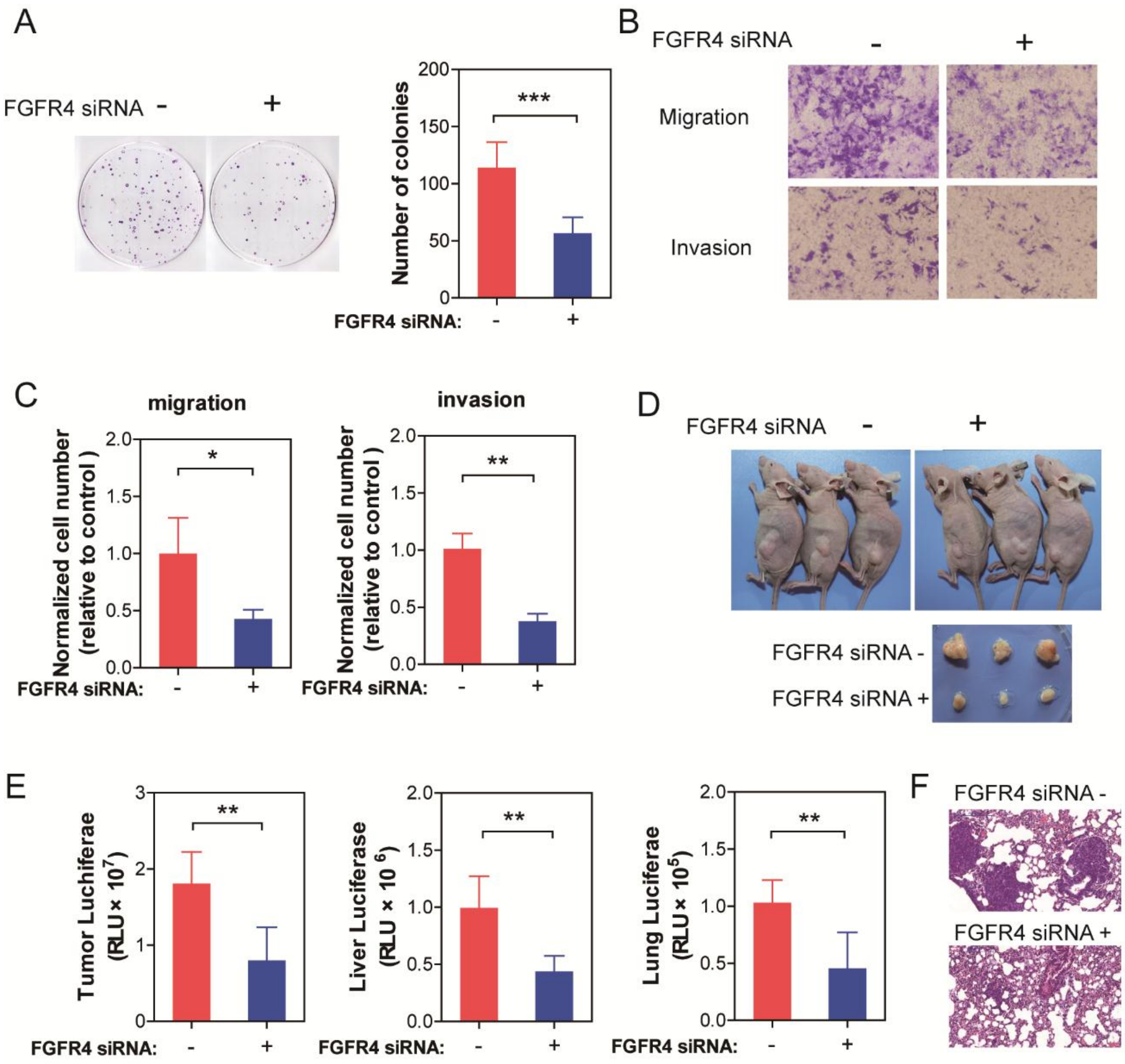

A
FGFR4 siRNA - +
Number of colonies
200
150
100
50
0
***
FGFR4 siRNA: - +
B
FGFR4 siRNA - +
Migration
Invasion
C
migration
invasion
Normalized cell number (relative to control)
2.0
1.5
1.0
0.5
0.0
*
**
FGFR4 siRNA: - +
D
FGFR4 siRNA - +
FGFR4 siRNA -
FGFR4 siRNA +
E
Tumor Luchiferae (RLU× 10^7)
Liver Luciferase (RLU × 10^6)
Lung Luciferae (RLU× 10^5)
**
FGFR4 siRNA: - +
F
FGFR4 siRNA -
FGFR4 siRNA +

Figure S4 Curcumin rescued mRNA expression to control levels in long-term PM-treated NCM460 cells.

A: Curcumin (20 μM) supplementation completely rescued FGFR4, PDGFB, CASP9 and SGK1 mRNA expression to control levels in long-term PM-treated NCM460 cells (*n*=6, two-way ANOVA). B: Relative to control, DHA (50 μM) supplementation partially rescued FGFR4 and CASP9 mRNA expression levels in long-term PM-treated NCM460 cells (*n*=6, two-way ANOVA). C: $1\alpha25\ (OH_2)\ D_3$ (100 nM) supplementation did not modulate mRNA expression levels in long-term PM-treated NCM460 cells (*n*=6, one-way ANOVA) * P<0.05, ** P<0.01, *** P<0.001).

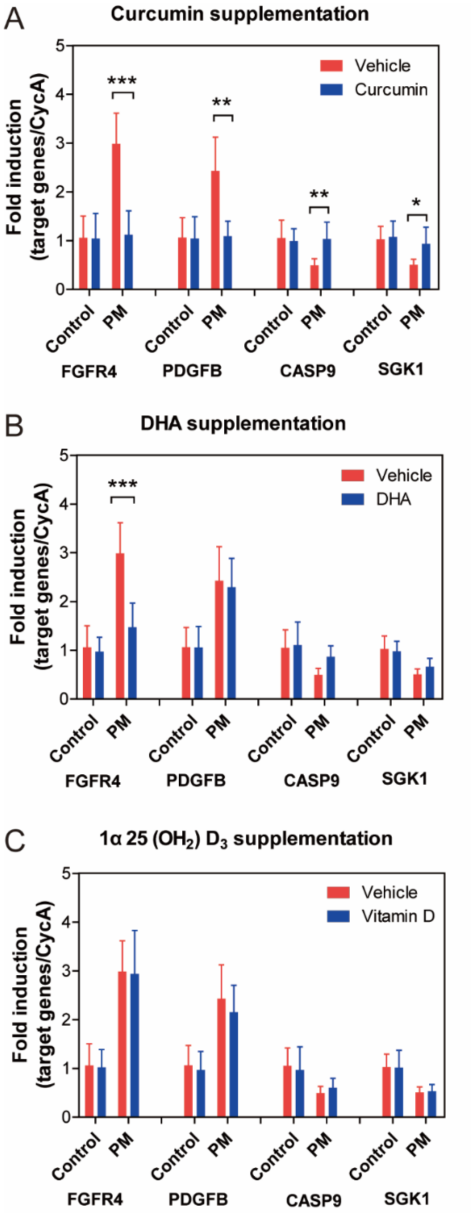

Table S1 Characteristics of subjects participating in the study [a)]

Variable	n	Mean	SD	min	25th	median	75th	max	IQR
Age	45	23.2	2.3	19.0	21.0	23.0	25.0	27.0	4.0
BMI	45	21.4	1.6	17.4	20.1	21.6	22. 5	25.3	2.3
IL-6	324	7.2	1.0	5.1	6.4	7.2	7.95	9.5	1.6
WBC/HPF	500	0.5	0.9	0.0	0.0	0.0	1.0	5.0	1.0

[a)] Abbreviations: BMI, body mass index; WBC, white blood cell; HPF, high power field

Table S2. Descriptive statistics of criteria air pollutants over the study period

Variable	n	Mean	SD	min	25th	median	75th	max	IQR
$PM_{2.5}$	57	84.1	58.1	12.0	36.0	63.0	119.0	256.0	83.0
PM_{10}	57	126. 7	72.6	18.0	69.0	116.0	166.0	347.0	97.0
NO_2	57	15.0	5.5	5.0	11.0	16.0	19.0	26.0	8.0
SO_2	57	63.1	23.9	25.0	42.0	64.0	83.0	126.0	41.0
CO	57	1.1	0.4	0.5	0.8	1.0	1.3	2.3	0.5
O_3	57	34.5	16.3	8.0	23.0	34.0	45.0	71.0	22.0

Table S3 Correlation analysis between six criteria air pollutants across the study period (Spearman r)

	**$PM_{2.5}$**	**PM_{10}**	**NO_2**	**SO_2**	**CO**	**O_3**
**$PM_{2.5}$**	1.00					
**PM_{10}**	**0.96**	1.00				
**NO_2**	0.32	**0.50**	1.00			
**SO_2**	**0.58**	**0.67**	**0.78**	1.00		
**CO**	**0.89**	**0.90**	**0.55**	**0.73**	1.00	
**O_3**	-0.20	-0.28	-0.40	**-0.60**	-0.39	1.00

Table S4 Cumulative increases in WBC/HPF measures associated with an IQR increase in air pollutant concentration

Air pollutant	Exposure metric	Estimate (95% confidence interval)	*P* value
**$PM_{2.5}$**			
	0-1 day	-0.18 (-0.49 to 0.31)	0.3964
	0-2 day	0.34 (-0.06 to 0.90)	0.1002
	0-3 day	0.39 (-0.01 to 0.96)	0.0552
	0-4 day	0.49 (0.13 to 0.96)	**0.0043**
	0-5 day	0.63 (0.22 to 1.19)	**0.0013**
	0-6 day	1.15 (0.38 to 2.33)	**0.0008**
	0-7 day	1.17 (0.23 to 2.81)	**0.0077**
	0-8 day	0.82 (0.05 to 2.15)	**0.0329**
	0-9 day	0.59 (-0.08 to 1.75)	0.0962
	0-10 day	0.80 (-0.02 to 2.30)	0.0587
**PM_{10}**			
	0-1 day	-0.16 (-0.47 to 0.33)	0.4661
	0-2 day	0.25 (-0.07 to 0.68)	0.1304
	0-3 day	0.33 (-0.02 to 0.79)	0.076
	0-4 day	0.48 (0.13 to 0.94)	**0.0054**
	0-5 day	0.62 (0.20 to 1.19)	**0.0021**
	0-6 day	1.10 (0.34 to 2.28)	**0.0015**
	0-7 day	1.48 (0.33 to 3.62)	**0.0046**
	0-8 day	1.12 (0.14 to 2.95)	**0.0183**
	0-9 day	0.82 (-0.07 to 2.56)	0.0780
	0-10 day	1.10 (-0.05 to 3.65)	0.0660
**NO_2**			
	0-1 day	-0.21 (-0.40 to 0.03)	0.0778
	0-2 day	-0.25 (-0.44 to 0.01)	0.0599
	0-3 day	-0.20 (-0.43 to 0.13)	0.2022
	0-4 day	-0.05 (-0.43 to 0.59)	0.8540
	0-5 day	0.15 (-0.34 to 0.98)	0.6204
	0-6 day	0.61 (-0.12 to 1.96)	0.1200
	0-7 day	0.77 (-0.04 to 2.27)	0.0654
	0-8 day	0.95 (-0.17 to 3.59)	0.1254
	0-9 day	0.47 (-0.50 to 3.37)	0.4821
	0-10 day	-0.13 (-0.95 to 1.55)	0.9249
**SO_2**			
	0-1 day	-0.18 (-0.44 to 0.20)	0.3076
	0-2 day	-0.19 (-0.47 to 0.24)	0.3299

	0-3 day	0.09 (-0.21 to 0.51)	0.5804
	0-4 day	0.23 (-0.16 to 0.79)	0.2849
	0-5 day	0.41 (-0.10 to 1.19)	0.1292
	0-6 day	0.85 (0.09 to 2.15)	**0.0238**
	0-7 day	1.64 (0.28 to 4.42)	**0.0089**
	0-8 day	3.91 (0.60 to 1.41)	**0.0059**
	0-9 day	2.26 (-0.51 to 2.08)	0.2204
	0-10 day	2.85 (-0.52 to 2.99)	0.2022
**CO**			
	0-1 day	0.18 (-0.23 to 0.82)	0.4345
	0-2 day	0.66 (0.13 to 1.42)	**0.0097**
	0-3 day	0.61 (0.20 to 1.16)	**0.0020**
	0-4 day	0.56 (0.16 to 1.09)	**0.0038**
	0-5 day	0.61 (0.17 to 1.20)	**0.0037**
	0-6 day	0.79 (0.17 to 1.74)	**0.0072**
	0-7 day	0.52 (-0.03 to 1.38)	0.0654
	0-8 day	0.54 (-0.05 to 1.49)	0.0769
	0-9 day	0.47 (-0.14 to 1.50)	0.1583
	0-10 day	0.72 (-0.02 to 2.00)	0.0569
**O_3**			
	0-1 day	0.15 (-0.08 to 0.44)	0.2282
	0-2 day	0.14 (-0.36 to 1.07)	0.6503
	0-3 day	-0.79 (-0.91 to 0.71)	**0.0027**
	0-4 day	-0.87 (-0.96 to -0.55)	**0.0015**
	0-5 day	-0.69 (-0.86 to -0.32)	**0.0041**
	0-6 day	-0.85 (-0.95 to -0.55)	**0.0009**
	0-7 day	-0.73 (-0.95 to 0.45)	0.1249
	0-8 day	-0.68 (-0.93 to 0.45)	0.1380
	0-9 day	-0.20 (-0.71 to 1.22)	0.6621
	0-10 day	-0.53 (-0.86 to 0.61)	0.2251

Table S5 The description of PI3K-AKT pathway involved genes

**Gene symbol**	**Description**
PIK3R3	phosphoinositide-3-kinase, regulatory subunit 3 (gamma)
CSF1R	colony stimulating factor 1 receptor
FGFR4	fibroblast growth factor receptor 4
SGK1	Serum/Glucocorticoid Regulated Kinase 1
CASP9	caspase 9, apoptosis-related cysteine peptidase
OSMR	oncostatin M receptor

PDGFB	platelet-derived growth factor beta polypeptide
ITGA5	integrin, alpha 5 (fibronectin receptor, alpha polypeptide)
PIK3CD	phosphatidylinositol-4,5-bisphosphate 3-kinase, catalytic subunit delta
CREB3L1	cAMP responsive element binding protein 3-like 1
TNXB	tenascin XB
FN1	fibronectin 1
IL4R	interleukin 4 receptor
VEGFC	vascular endothelial growth factor C
CCND2	cyclin D2
F2R	coagulation factor II (thrombin) receptor
COL6A1	collagen, type VI, alpha 1
COL6A2	collagen, type VI, alpha 2
FGF18	fibroblast growth factor 18
COL3A1	collagen, type III, alpha 1
TNC	tenascin C
COL4A4	collagen, type IV, alpha 4

Table S6 The sequences of primers used for qRT-PCR assay

**Gene symbol**	**Forward**	**Reverse**
hPDGFB	5’CTCGATCCGCTCCTTTGATGA3’	5’CGTTGGTGCGGTCTATGAG3’
hFGFR4	5’GCACTGGAGTCTCGTGATGG3’	5’CCACAGCGTTCTCTACCAGG3’
hSGK1	5’AGGATGGGTCTGAACGACTTT3’	5’GCCCTTTCCGATCACTTTCAAG3’
hCASP9	5’CTCAGACCAGAGATTCGCAAAC3’	5’GCATTTCCCCTCAAACTCTCAA3’
mp65	5’AGGCTTCTGGGCCTTATGTG3’	5’TGCTTCTCTCGCCAGGAATAC3’
mStat3	5’CAATACCATTGACCTGCCGAT3’	5’GAGCGACTCAAACTGCCCT3’
mRelB	5’CCGTACCTGGTCATCACAGAG3’	5’CAGTCTCGAAGCTCGATGGC3’
mNrf2	5’CTGTTCGGGAGCGTGGAAA3’	5’CAGTAGCAAACTTGCCCCCTT3’
